# Targeting Anticipatory Neurogenesis to Maintain Cognitive Reserve

**DOI:** 10.1093/geroni/igab046.757

**Published:** 2021-12-17

**Authors:** Amar Sahay

**Affiliations:** Massachusetts General Hospital, Harvard Medical School, Lexington, Massachusetts, United States

## Abstract

Memory imprecision is a hallmark of age-related cognitive decline and mild-cognitive impairment (MCI) and is characterized by increased memory interference and decreased stability of memory representations. Evidence from humans, non-human primates and rodents demonstrate reduced hippocampal neurogenesis, excitation-inhibition imbalance and inflexible hippocampal remapping during age-related cognitive decline and MCI. Developing strategies to reverse cognitive decline during aging and Mild Cognitive Impairment necessitates an understanding of molecular, cellular, circuit and network mechanisms that support memory functions of the hippocampus. Over the last decade we have built a multifaceted program grounded in basic neuroscience that is aimed at improving memory in aging and MCI. We have demonstrated how we can Rejuvenate the aged hippocampus by selectively increasing neurogenesis and how we can Re-engineer connectivity of aged inhibitory microcircuits to improve memory precision in aging. Ongoing efforts include strategies to Repairing neurogenic niche fitness by targeting intercellular communication in the aging hippocampus. In today’s talk I will present a fourth approach catalyzed by our discovery of the first transcriptional regulator of neural stem cell expansion in the adult hippocampus. We will present data in support of this claim and convey how this discovery may guide strategies to maintain cognitive reserve embodied in the pool of neural stem cells in the adult hippocampus.

